# The Effectiveness of Infliximab in Treating Vascular Manifestations of Behçet’s Disease: A Systematic Review and Meta-Analysis

**DOI:** 10.31138/mjr.080325.tav

**Published:** 2025-08-26

**Authors:** Aymane Bennani, El Khalil Benmansour, Khalid Serraj

**Affiliations:** 1 Department of Internal Medicine, University Hospital Mohammed VI Oujda, Morocco,; 2 Immunology and Cellular Therapy Laboratory, Faculty of Medicine and Pharmacy, Mohammed First University Oujda, Morocco

**Keywords:** Infliximab, Behçet Syndrome, vascular diseases, meta-analysis

## Abstract

**Objective:**

Our study aims to evaluate the effectiveness of anti-tumour-necrosis-factor Infliximab (IFX) in treating patients with severe and/or refractory vascular manifestations of Behçet’s disease (BD) through a systematic review and meta-analysis.

**Methods:**

We searched PubMed, Scopus, Web of Science, and Cochrane Library databases for eligible studies. Meta-analysis of proportions was performed. Heterogeneity was assessed with Cochrane’s Q test and I^2^ statistics. Random effects models were used for all statistical analyses which were conducted on R software (version 4.4.2), PROSPERO registry number CRD42025640970*.*

**Results:**

Of the 2127 results initially identified, 11 studies with a total of 284 patients were included in the final analysis. Infliximab induced complete vascular response in 78.17% of patients (95% CI 63.11–90.61). The remission rates in the subgroups of patients with pulmonary artery aneurysm and the venous thrombosis events were respectively 99.13% (95% CI 86.21–100) and 86.48 % (95% CI 71.81–97.15).

**Conclusion:**

Our systematic review and meta-analysis support the use of infliximab in patients with severe and/or refractory vascular Behçet’s disease, particularly in cases involving pulmonary artery aneurysms.

## INTRODUCTION

Behçet’s disease (BD) is a chronic and relapsing disorder, characterised by diverse multisystemic clinical features including mucocutaneous, ocular, vascular, articular, gastrointestinal, and neurological manifestations.^1^ The pathophysiology of BD is not yet fully elaborated, but an association with genetic factors (such as HLA-b51) was highlighted, and autoinflammatory and autoimmune features have been described; ^2^ as several reports have suggested an adaptive immune response mediated by CD4+T-helper lymphocytes, which after differentiation induce the production of proinflammatory cytokines (e.g., tumour necrosis factor α, interferon-γ, Interleukin 17 and 23), cytotoxic T-cell activity, and the promotion of neutrophil chemotaxis.^2^

Vascular involvement is seen in up to 40% of BD patients.^3^ The first vascular incident occurs within 5 years of disease onset in 75% of patients,^4^ and can in 28% of cases be the first manifestation of the disease or even precede the diagnosis of BD.^5^ Vascular BD affects both arterial and venous systems across various calibers.^5^ Venous thrombotic events occur more frequently than arterial complications, with deep vein thrombosis (DVT) being the most commonly observed vascular feature and arterial disease mainly manifesting as aneurysms.^3^ Major vessel involvement is also the leading cause of mortality in BD,^6^ and arterial involvement has been shown to be independently associated with the risk of mortality,^6^ hence the importance of managing Behçet’s-related vasculopathy.

Current guidelines from the European League Against Rheumatism (EULAR) recommend the use of immunosuppressants (e.g., corticosteroids, cyclophosphamide, azathioprine, and cyclosporine-A)^7^ based on the type of lesion, but more than 30% of patients continue to experience recurrent vascular events despite treatment. ^4,8^ Anti-tumour-necrosis factor (anti-TNF) alpha monoclonal antibodies (namely infliximab) are increasingly explored as therapeutic alternatives, particularly for severe and refractory cases, and while previous systematic reviews and meta-analyses have assessed the efficacy of infliximab (IFX) in managing neurological,^9,10^ intestinal,^11,12^ and ocular^13,14^ manifestations of BD, none has specifically examined its role in vascular complications. Thus, we conducted a systematic review and meta-analysis of previously published studies to address this gap and evaluate the effectiveness of infliximab in the treatment of vascular BD.

## METHODS

This systematic review and meta-analysis was conducted in accordance with the recommendations of the Cochrane Collaboration Handbook for Systematic Review of Interventions and the Preferred Reporting Items for Systematic Reviews and Meta-Analyses (PRISMA) Statement guidelines,^15^ and it was registered in the International prospective register of systematic reviews PROSPERO under the protocol number CRD42025640970.

### Search strategy and data extraction

The search strategy was developed and reported in line with the recommendations endorsed by the Mediterranean Journal of Rheumatology^16^ and the PRISMA Statement guidelines.^15^

PubMed, Scopus, Web of Science, and Cochrane Library databases were systematically searched for eligible studies. The search strategy included the following terms and Boolean operators: “Behçet” OR “Behçet syndrome” OR “Vascular Behçet”; AND infliximab OR IFX; AND “vascular diseases” OR vascular OR arterial OR venous OR thrombosis OR aneurysm OR vasculitis. Eligible criteria for inclusion were: (i) studies investigating patients: 1- diagnosed with severe or refractory Behçet’s disease, 2- with vascular manifestations (arterial or venous) reported and followed up, 3- treated with monoclonal anti-tumour-necrosis-factor antibody Infliximab, (ii) randomised controlled trials, non-randomised controlled trials or observational studies (prospective or retrospective) as study design. Studies involving patients receiving other anti-TNF therapies, those with missing vascular manifestations or outcomes, studies with overlapping populations, case reports, reviews, and conference abstracts were excluded.

Two authors (AB and EKB) independently screened the records based on title and abstract, selected papers to be read fully and then manually extracted data from included studies. Disagreements were resolved by consensus or after consulting with a third author (KS). We collected data on study design, follow-up duration, the number of patients with vascular events treated with infliximab, infliximab dosage, and the different vascular manifestations reported in the infliximab group. The primary endpoint was the achievement of Complete Vascular response (CVr) after infliximab therapy, defined as the absence of any newly emergent clinical or radiological vascular manifestation of Behçet’s disease and no progression of pre-existing lesions. Two subgroup analyses were conducted to evaluate remission rates in patients with pulmonary artery aneurysm (PAA) and the venous thrombosis events.

### Risk of bias assessment

The Cochrane risk-of-bias tool for randomised trials (RoB 2)^17^ was used to assess bias in the included randomised trial and the ROBINS-I tool (“Risk Of Bias In Non-randomised Studies of Interventions”)^18^ was used to evaluate bias in the included other non-randomised, non-controlled studies.

### Statistical analysis

R software (version 4.4.2) was used for all statistical analyses. A proportional meta-analysis was conducted for the primary outcome and the subgroups analyses, results were represented as proportions with 95% confidence intervals (CI). A double-arcsine transformation was applied when a study reported a proportion of 1. Heterogeneity was assessed using Cochrane’s Q test and I^2^ statistics. A leave-one-out analysis of the primary endpoint was performed to investigate the high interstudy heterogeneity.

## RESULTS

### Study selection and characteristics of included studies

**Figure 1.**
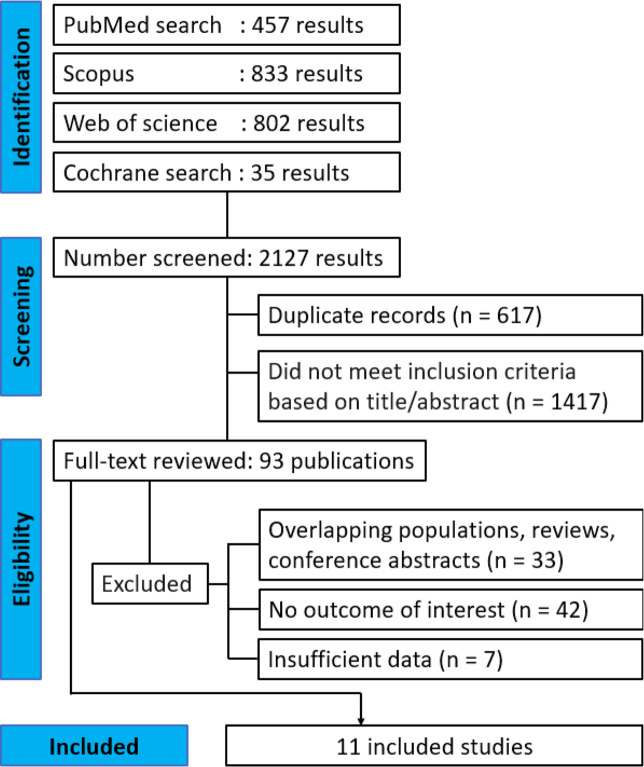
PRISMA flow diagram of study screening and selection.

The search strategy identified a total of 2127 results (**Figure 1**). After removing duplicates and screening titles and abstracts, 93 publications were retrieved for full-text review. Of these, 11 studies met all the inclusion criteria and were included in the final analysis.

The included studies yielded a total of 284 patients with vascular manifestations of Behçet’s disease treated with infliximab. Infliximab was mainly administered at a dose of 5mg/kg IV. In the study by Hibi et al. 2024,^19^ an escalation to 10mg/kg was allowed for patients who experienced an inadequate or loss of response. In the study by Adler et al. 2012,^20^ the choice of dose was stratified according to the anatomical distribution of the lesions (3mg/kg for peripheral vasculitis and 5 mg/kg for central vasculitis). In the study by Desbois et al. 2018, ^21^ the choice between 3mg/kg and 5mg/kg was not explained, likely due to the retrospective collection of the data. Other characteristics of the studies, including follow-up duration, study design, and reported vascular events, are summarised in **Table 1**.

**Table 1.** Characteristics of included studies.

	**Type of study**	**Follow-up Time (Range)**	**Dose (mg/kg IV)**	**N°V**	**Arterial events**			**Venous events**			**ICT**
					Aneurysm	Thrombosis or stenosis	Other	IVCT	DVT	CVT	
Saadoun et al. 2024^22^	RCT	22 weeks ^a^	5	19	7	3	-	-	11	1	-
Kehribar et al. 2020^33^	Retrospective	39 months ^§^	5	18	4 (2 PAA)	-	-	6	1	7	-
Aksoy et al. 2020^34^	Retrospective	14 months ^§^ (3–67)	5	24	8 (3PAA)	6(5PAT)	-	2	7	2	3
Hibi et al. 2024^19^	Prospective	2 years	5–10	51	-	-	-	-	-	-	-
Hibi et al. 2016^31^	Prospective	377.3 days ^µ^ (374–380)	5	4	-	1	-	-	1	-	-
Vallet et al. 2015^23^	Retrospective	21 months ^§^	5	4	-	-	-	-	-	-	-
Hatemi et al. 2023^35^	Retrospective	26 months ^§^ (6–105)	5	127	59 (12 PAA and 25 PAT)		-	8	44	18	11
Giardina et al. 2009^24^	Prospective	1 year	5	5	-	-	-	-	-	5	-
Desbois et al. 2018^21^	Retrospective	15 months ^§^	3 - 5	15	9 (4 PAA)	5(all PAT)	-	4	-	-	2
Hamuryudan et al. 2015^25^	Retrospective	19 months ^§^ (9–51 months)	5	12	5 (all PAA)	9 (all PAT)	-	2	8	3	5
Adler et al. 2012^20^	Retrospective	9 months to several years	3 -5	5	-	1	4 ^b^	-	2	-	-

a: (additional follow up to 3 years of observational period);

b: aortic dissection, aortitis, internal carotid artery dissection, endocarditis aortic;

§: Median;

µ: Mean;

N°V: number of patients with vascular events treated with infliximab; IVCT: inferior vena cava thrombosis; DVT: deep vein thrombosis; CVT: Cerebral vein vasculitis and/or thrombosis; ICT: intracardiac thrombosis; RCT: Randomised controlled trial; PAA: pulmonary artery aneurysm; PAT: Pulmonary artery thrombosis;(-) : no mention or occurrence of the event.

### Efficacy outcomes

**Figure 2.**
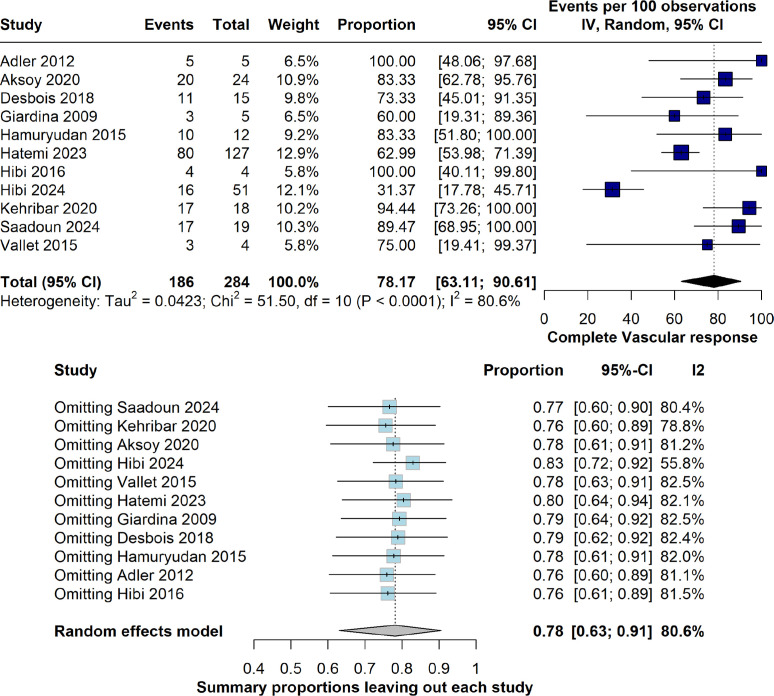
**(A)** Forest plot summarising meta-analysis results of the effectiveness of IFX in treating vascular BD. Overall, 78.17% of patients achieved (CVr), as indicated by the black diamond. Blue squares represent study weight, and the horizontal lines crossing them indicate the 95% confidence intervals. Events: patients who responded to IFX; Total: number of patients with vascular BD treated with IFX. **(B)** Leave-one-out sensitivity analysis illustrating the impact of sequentially excluding each study on the overall proportion of patients achieving Complete Vascular response, as well as its influence on interstudy heterogeneity.

The pooled analysis of the included studies showed that 78.17% of patients achieved Complete Vascular response (CVr) after infliximab treatment (95% CI 63.11–90.61) (**Figure 2A**). the Cochrane’s Q test (P<0.0001) and I^2^=80.6% revealed high interstudy heterogeneity, which we explored by performing a leave-one-out analysis (**Figure 2B**). The overall proportion of patients achieving (CVr) increased to 83% (95% CI 0.72–0.92) after excluding the study by Hibi et al. 2024,^19^ with a reduction of heterogeneity (I^2^ decreased to 55.8%).

**Figure 3.**
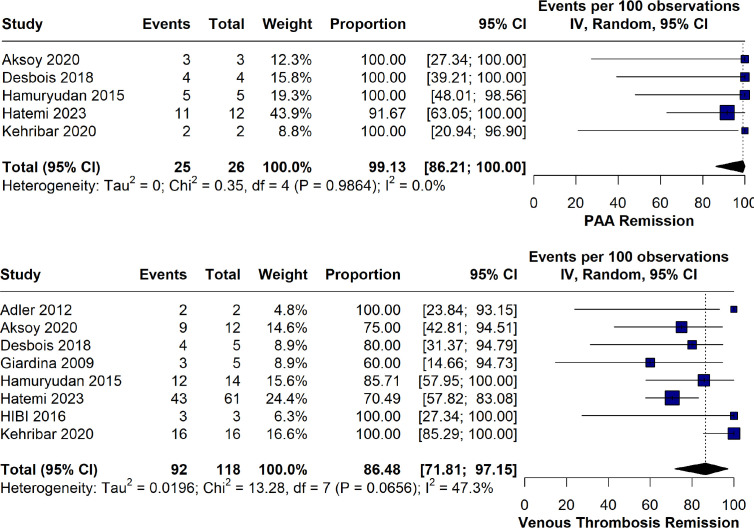
**(A)** Forest plot summarising the meta-analysis findings on the efficacy of infliximab for achieving remission in patients with pulmonary artery aneurysm (PAA). The blue squares represent the relative weight of each study and the horizontal lines extending from them indicate the corresponding 95% confidence intervals. **(B)** Forest plot summarising the meta-analysis findings on the efficacy of infliximab in inducing remission in venous thrombosis events. The blue squares represent the relative weight of each study and the horizontal lines extending from them indicate the corresponding 95% confidence intervals. Events: the number of venous thrombosis incidents resolved with IFX treatment, Total: the total number of venous thrombosis incidents among patients treated with IFX.

The leave-one-out analysis also exhibited consistent results across the remaining studies, thus reinforcing the results of our meta-analysis. The response rates based on study design and bias are available in the supplementary material. In the subgroup analysis, patients with pulmonary artery aneurysm (PAA) exhibited a good response to infliximab. The overall remission rate of PAA events was 99.13 % (95 %CI 86.21–100) and the interstudy heterogeneity was not statistically significant (P=0.9684; I^2^=0%) (**Figure 3A**). In addition, 86.48 % of venous thrombosis events responded to infliximab (95% CI 71.81–97.15), (P=0.0502; I^2^=47.3%) (**Figure 3B**).

### Risk of bias assessment

Rob 2 tool ^17^ was used to evaluate the risk of bias in the study by Saadoun et al. 2024 ^22^ as it was a randomised controlled trial (**Table 2A**). The assessment identified some concerns due to the absence of blinding and the lack of detailed statistical adjustments for the impact of co-interventions (e.g., corticosteroids) on the reported outcomes.

**Table 2A:** Risk of bias summary for randomised studies (RoB 2)

**Study**	**Bias from randomisation process**	**Bias due to deviations from intended interventions**	**Bias due to missing outcome data**	**Bias in measurement of the outcomes**	**Bias in selection of the reported result**	**Overall risk of bias**
Saadoun et al. 2024^22^	Low	Some concerns	Low	Some concerns	Some concerns	Some concerns

The risk of bias in non-randomised, non-controlled studies was assessed using the ROBINS-I tool. ^18^ Given that the majority of these studies were observational, they did not adequately adjust for potential confounding factors. Three studies^20,23,24^ were considered to have serious risk of selection bias due to the absence of clearly defined objective criteria or diagnostic tests for vascular manifestations of Behçet’s disease. Four others^20,21,24,25^ were identified as having serious risk of bias in measurement of outcomes, as they failed to predefine objective measurements of vascular outcomes in the methods section before collecting the data. Studies were deemed to have a high risk of reporting bias if they did not specify a priori which outcomes would be reported in the methods section before presenting the results. The study by Desbois et al. 2018 ^21^ exhibited the highest risk of bias, as discrepancies were identified between the results reported in the text and the corresponding data presented in the tables. A detailed appraisal of each study is provided in (**Table 2B**).

**Table 2B:** Risk of bias summary for non-randomised studies (ROBINS-I).

**Study**	**Bias due to confounding**	**Bias in selection of participants**	**Bias in classification of interventions**	**Bias due to deviations from intended interventions**	**Bias due to missing data**	**Bias in measurement of outcomes**	**Bias in selection of the reported result**	**Overall risk of bias judgement**
Kehribar et al. 2020^33^	Moderate	Moderate	Low	low	Low	Moderate	Moderate	Moderate
Aksoy et al. 2020^34^	Moderate	Moderate	Low	Low	Low	Moderate	Moderate	Moderate
Hibi et al. 2024^19^	Moderate	Moderate	Low	Low	Serious	Moderate	Moderate	Moderate
Hibi et al. 2016^31^	Moderate	Moderate	Low	Low	Moderate	Moderate	Moderate	Moderate
Vallet et al. 2015^23^	Moderate	Serious	Low	Low	Low	Moderate	Moderate	Moderate
Hatemi et al. 2023^35^	Moderate	Moderate	Low	Low	Low	Moderate	Moderate	Moderate
Giardina et al. 2009^24^	Moderate	Serious	Low	Low	Moderate	Serious	Moderate	Moderate
Desbois et al. 2018^21^	Moderate	Moderate	Low	Low	Serious	Serious	Serious	Serious
Hamuryudan et al. 2015^25^	Moderate	Moderate	Low	Low	Low	Serious	Serious	Moderate
Adler et al. 2012^20^	Moderate	Serious	Low	Low	Low	Serious	Serious	Moderate

## DISCUSSION

Behçet’s disease (BD) is a multifactorial disorder that manifests in genetically predisposed individuals following exposure to environmental or infectious triggers. From a pathophysiological perspective, all manifestations of Behçet’s disease share an underlying inflammatory and dysregulated immune response. However, vascular involvement in Behçet’s disease is distinguished by additional mechanisms of endothelial dysfunction and hypercoagulability. ^26^ This distinctive pathophysiology implies that the therapeutic efficacy of Anti-tumour-necrosis factor (anti-TNF)-α monoclonal antibodies observed in other manifestations of Behçet’s disease^9–14^ cannot be directly extrapolated to its vascular complications. Existing studies that explored anti-TNF-α agents as potential treatment for vascular BD are scarce and were not synthesised, so no conclusions were drawn and recommendations remained cautious.^7^ We selected infliximab for our study as it is the most studied and frequently used anti-TNF-α agent in the context of BD.^27^

Infliximab was initially approved by the US Food and Drug Administration (FDA) in 1998 for the treatment of Crohn’s disease, then the indication expanded to other inflammatory conditions like rheumatoid arthritis, ankylosing spondylitis, psoriatic arthritis, and ulcerative colitis.^28^ Its consideration for the management of BD started in the early 2000, mainly for the treatment of uveitis in BD patients,^29^ in which it showed promising results. Over time, more studies explored the efficacy of infliximab in the other manifestations of BD including vascular BD as the vascular complications have important repercussions on psychological and social aspects of BD patients, and are acute and life-threatening.^30^ Conventional immunosuppressive therapies, considered first-line treatment,^7^ typically require several weeks to achieve therapeutic efficacy, a delay that can be critical for patients with vascular involvement. In this context, the study by Adler et al. 2012,^20^ one of the earliest studies to investigate infliximab for vascular BD, showed rapid control of systemic inflammation with five days for all patients after infliximab infusion. A remarkable and fast result, however the study was retrospective and only included 7 patients. Therefore, our systematic review and meta-analysis aimed to address this limitation by providing a comprehensive and evidence-based analysis.

In our systematic review and meta-analysis of 11 studies, 78.17% of 284 patients achieved Complete Vascular response (CVr) after infliximab therapy (CVr was defined as the absence of any newly emergent clinical or radiological vascular manifestation of Behçet’s disease and no progression of pre-existing lesions). The Cochrane’s Q test (P<0.0001) and I^2^ statistics (=80.6%) revealed high interstudy heterogeneity, which was expected, as the included studies had different study design (1 RCT, 3 prospective, and 7 retrospective studies), different inclusion criteria (some studies included patients with severe BD manifestations in general and then reported vascular outcomes particularly,^19,22,31^ while others only included patients with specific vascular manifestations of BD, like major vessel involvement ^21,25^), different times of follow-up, and sometimes different IFX dosage (3-5-10 mg/kg). We tried to explore the heterogeneity further by performing a leave-one-out analysis that showed an increase in the overall proportion of patients achieving (CVr) to 83% (95% CI 0.72–0.92) after excluding the study by Hibi et al. 2024,^19^ with a reduction in heterogeneity (I2 decreased to 55.8%). This could be due to the fact that out of the 51 patients with vascular BD included in the study by Hibi et al. 2024,^19^ only 16 patients achieving CVr made it to the final evaluation, as the number of patients with vascular BD fluctuated throughout the follow-up, and data regarding the reasons for loss to follow-up were not clearly stated, thus making it the study with the lowest proportion in the analysis (31.37%). The study by Hibi et al. 2024^19^ was also the prospective trial with the most patients, while the majority of the included studies were retrospective. In addition, the study did not provide detailed stratification of vascular lesion types and only reported the total number of patients with arterial and/or venous involvement. Furthermore, imaging data were incomplete, in contrast to more comprehensive imaging details in the other studies. All of these reasons might have contributed to the study’s outlier status within the meta-analysis. Importantly, the leave-one-out analysis exhibited consistent results across the remaining studies, thereby consolidating the results of our meta-analysis.

Since pulmonary artery aneurysm (PAA) is one of the main causes of death in BD,^6^ we conducted a subgroup analysis for PAA patients that showed remarkable results. The overall remission was 99.13 % (95 %CI 86.21–100) and the interstudy heterogeneity was not statistically significant (P=0.9684; I^2^=0%). This finding may suggest more use of infliximab in the context of PAA, while waiting for further studies. Venous thrombosis events also responded well to IFX (86.48%, 95% CI 71.81–97.15), a promising finding given that venous thromboses are among the most prevalent vascular manifestations of BD.^4,32^

We evaluated the risk of bias using the Rob 2 tool in the study by Saadoun et al. 2024^22^ as it was a randomized controlled trial. The overall evaluation indicated some concerns mainly due to the absence of blinding and insufficiency of statistical adjustments for the impact of co-interventions (e.g., corticosteroids) on the reported outcomes. The ROBINS-I tool was used in the remaining observational studies. Although the observational and non-controlled design of studies may introduce potential biases, they provide real-world insights that can be difficult to replicate in the controlled environment of a randomized controlled trial. Furthermore, certain vascular complications associated with Behçet’s disease are relatively rare, and retrospective observational studies remain a practical approach to accumulating sufficient case data for meaningful analysis.

Our systematic review and meta-analysis revealed important limitations within the literature, as there is still a lack of well-designed, high-quality studies specifically investigating infliximab in the treatment of vascular BD. Patients with vascular involvement are underrepresented and often included within cohorts investigating other manifestations of BD. In addition, patients treated with infliximab for vascular BD can receive concomitantly immunosuppressants agents. In the study by Kehribar et al. 2020^33^ for example, nine patients were receiving azathioprine concomitantly with Infliximab and in the study by Aksoy et al. 2020,^34^ more patients reached complete remission with concomitant IS use (93%) compared to anti-TNFα monotherapy (67%). In the study by Vallet et al. 2015,^23^ anti-TNFα agents even had a significant corticosteroid-sparing effect. However, robust evidence highlighting the role of concomitant immunosuppressive therapy, including optimal dosing strategies and treatment algorithms for vascular BD patients, is still lacking. Moreover, further investigation is needed to examine the perioperative use of infliximab and its potential influence on surgical outcomes, as the management of some vascular complications may require surgery. The use of anticoagulants also remains controversial. The EULAR recommendations^7^ mainly addressed the association between anticoagulants and immunosuppressants; the concomitant use of anti-TNFα agents and anticoagulants is still unclear. Finally, while infliximab showed promising results, data regarding safety effects among vascular BD subgroups are limited. In the study by Hibi et al. 2024,^19^ 6 vascular BD patients had serious infections and in the study by Hatemi et al. 2023,^35^ 19 patients had serious adverse side events that included infusion reaction, infectious events (tuberculosis / disseminated zona), and neoplasia (lung adenocarcinoma / fibromyxoid sarcoma). Since the acceptance of our manuscript on 6th May 2025, a recent systematic review and meta-analysis examining class-level effects of monoclonal anti-TNF-α agents on vascular BD has been published.^36^ Although several primary studies overlap, key elements of distinction include: (i) their scope was class-level, whereas our review isolates infliximab, reducing intervention heterogeneity and yielding drug-specific estimates. Notably, the class-level review reported infliximab-only subgroup, however, that analysis drew on fewer contributing studies than ours and adopted different follow-up intervals. (ii) Our outcome choices diverged (they reported time-based response while we conducted subgroup analyses for patients with PAA and venous thrombosis events), (iii) Methodological approaches also differed (we opted for distinct sensitivity analyses and tools for risk of bias assessment). Therefore, our findings complement the class-level synthesis by providing drug-specific guidance.

## CONCLUSION

The authors of this systematic review and meta-analysis support the use of infliximab in patients with severe and/or refractory vascular Behçet’s disease, particularly in cases involving pulmonary artery aneurysms, a leading cause of mortality in BD. The authors also recommend future studies in the field to address the highlighted limitations.

## Figures and Tables

**Supplementary Figure S1.**
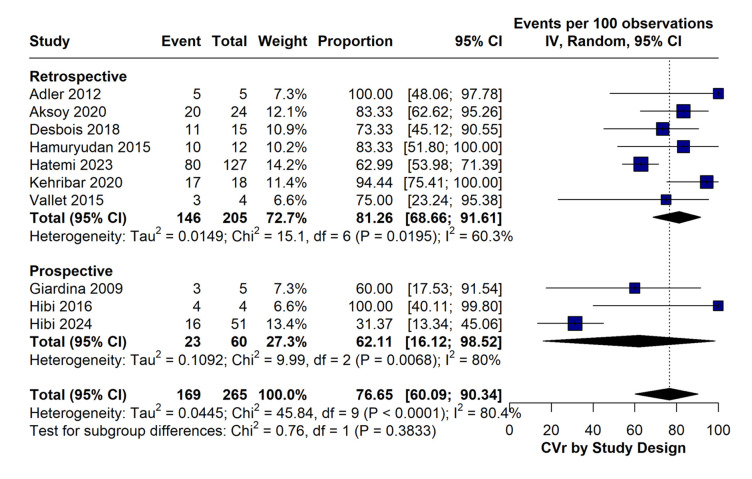
Forrest plot summarising meta-analysis results of the primary outcome (CVr) based on study design. Retrospective studies showed higher response rate (81.26% vs 76.65%) and less interstudy heterogeneity (60.3% vs 80%) than prospective studies.

**Supplementary Figure S2.**
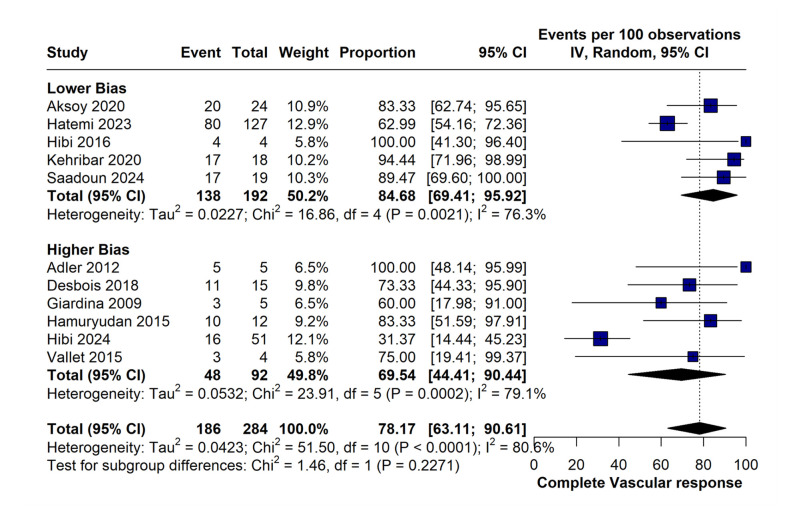
Forrest plot summarising meta-analysis results of the primary outcome (CVr) based on Bias. Studies with lower bias showed higher complete vascular response rate than high bias studies (84,68% vs 69,54%). The interstudy heterogeneity did not differ much (76.3% for lower bias studies vs 79.1% for higher bias studies).

## OPEN DATA SHARING NOTE

All the data we used in our analysis are publicly accessible in the published studies cited in the reference list.

## References

[1] Saadoun D Wechsler B. Behçet’s disease. Orphanet J Rare Dis 2012;7:20. doi:10.1186/1750-1172-7-20 22497990 PMC3472229

[2] Saadoun D Bodaghi B Cacoub P. Behçet’s syndrome. N Engl J Med 2024;390(7):640–51. doi:10.1056/NEJMra2305712 38354143

[3] Seyahi E. Phenotypes in Behçet’s syndrome. Intern Emerg Med 2019;14(5):677–89. doi:10.1007/s11739-019-02046-y 30747365

[4] Tascilar K Melikoglu M Ugurlu S Sut N Caglar E Yazici H. Vascular involvement in Behçet’s syndrome: a retrospective analysis of associations and the time course. Rheumatol Oxf Engl 2014;53(11):2018–022. doi:10.1093/rheumatology/keu233 24907156

[5] Alibaz-Oner F Vautier M Aksoy A Mirouse A Le Joncour A Cacoub P Vascular Behçet’s disease: a comparative study from Turkey and France. Clin Exp Rheumatol 2022;40(8):1491–6. doi:10.55563/clinexprheumatol/iovig5 35200121

[6] Saadoun D Wechsler B Desseaux K Le Thi Huong D Amoura Z Resche-Rigon M Mortality in Behçet’s disease. Arthritis Rheum 2010;62(9):2806–12. doi:10.1002/art.27568 20496419

[7] Hatemi G Christensen R Bang D Bodaghi B Celik AF Fortune F 2018 update of the EULAR recommendations for the management of Behçet’s syndrome. Ann Rheum Dis 2018;77(6):808–18. doi:10.1136/annrheumdis-2018-213225 29625968

[8] Desbois AC Wechsler B Resche-Rigon M Piette JC Huong Dle T Amoura Z Immunosuppressants reduce venous thrombosis relapse in Behçet’s disease. Arthritis Rheum 2012;64(8):2753–60. doi:10.1002/art.34450 22354392

[9] Mohammed RHA Woldeamanuel YW. The effectiveness of the anti-tumor necrosis factor therapy infliximab in neuro-Behçet’s disease: a systematic review and meta-analysis. J Int Med Res 2023;51(5):3000605231169895. doi:10.1177/03000605231169895 37203384 PMC10201531

[10] Wang X Liu Y Ao Y Wang J Liu J Xu Y Infliximab for parenchymal neuro-Behçet’s syndrome: case series and meta-analysis. Clin Exp Rheumatol 2024;42(10):2040–8. doi:10.55563/clinexprheumatol/5lnkd4 38757293

[11] Zhan S Liu C Li N Li T Tian Z Zhao M Anti-TNF-α agents for refractory intestinal Behçet’s disease: case series and meta-analysis. Ther Adv Gastroenterol 2022;15. doi:10.1177/17562848221116666 PMC9445467 36082178

[12] Zhang M Liu J Liu T Han W Bai X Ruan G The efficacy and safety of anti-tumor necrosis factor agents in the treatment of intestinal Behçet’s disease, a systematic review and meta-analysis. J Gastroenterol Hepatol 2022;37(4):608–19. doi:10.1111/jgh.15754 34894004

[13] Guan X Zhao Z Xin M Xia G Yang Q Fu M. Long-term efficacy, safety, and cumulative retention rate of antitumor necrosis factor-alpha treatment for patients with Behçet’s uveitis: A systematic review and meta-analysis. Int J Rheum Dis 2024;27(2):e15096. doi:10.1111/1756-185X.15096 38402428

[14] Abolhasani S Khabbazi A Hosseini F Aziz S Alipour S. Effects of anti-TNF biologic drugs on uveitis severity in Behçet patients: systematic review and Meta-analysis. Int J Ophthalmol 2022;15(5):813–9. doi:10.18240/ijo.2022.05.19 35601158 PMC9091884

[15] Page MJ McKenzie JE Bossuyt PM Boutron I Hoffmann TC Mulrow CD The PRISMA 2020 statement: an updated guideline for reporting systematic reviews. The BMJ 2021;372:n71. doi:10.1136/bmj.n71 33782057 PMC8005924

[16] Gasparyan AY Ayvazyan L Blackmore H Kitas GD. Writing a narrative biomedical review: considerations for authors, peer reviewers, and editors. Rheumatol Int 2011;31(11):1409–17. doi:10.1007/s00296-011-1999-3 21800117

[17] Sterne JAC Savović J Page MJ Elbers RG Blencowe NS Boutron I RoB 2: a revised tool for assessing risk of bias in randomised trials. BMJ 2019;366:l4898. doi:10.1136/bmj.l4898 31462531

[18] Sterne JA Hernán MA Reeves BC Savović J Berkman ND Viswanathan M ROBINS-I: a tool for assessing risk of bias in non-randomised studies of interventions. BMJ 2016;355:i4919. doi:10.1136/bmj.i4919 27733354 PMC5062054

[19] Hibi T Hirohata S Hisamatsu T Kikuchi H Takeno M Sato N Real-World Safety and Effectiveness of Infliximab in 255 Patients with Intestinal, Neurological, and Vascular Behçet’s Disease: A Post-Marketing Surveillance. Adv Ther 2024;41(12):4476–97. doi:10.1007/s12325-024-02993-9 39412631 PMC11550226

[20] Adler S Baumgartner I Villiger PM. Behçet’s disease: Successful treatment with infliximab in 7 patients with severe vascular manifestations. A retrospective analysis. Arthritis Care Res 2012;64(4):607–11. doi:10.1002/acr.21557 22162223

[21] Desbois AC Biard L Addimanda O Lambert M Hachulla E Launay D Efficacy of anti-TNF alpha in severe and refractory major vessel involvement of Behçet’s disease: A multicenter observational study of 18 patients. Clin Immunol 2018;197:54–9. doi:10.1016/j.clim.2018.08.004 30125675

[22] Saadoun D Maalouf G Vieira M Trad S Lazaro E Sacre K Infliximab versus Cyclophosphamide for Severe Behçet’s Syndrome. NEJM Evid 2024;3(11):EVIDoa2300354. doi:10.1056/EVIDoa2300354 39437137

[23] Vallet H Riviere S Sanna A Deroux A Moulis G Addimanda O Efficacy of anti-TNF alpha in severe and/or refractory Behçet’s disease: Multicenter study of 124 patients. J Autoimmun 2015;62:67–74. doi:10.1016/j.jaut.2015.06.005 26162757

[24] Giardina A Ferrante A Ciccia F Vadalà M Giardina E Triolo G. One year study of efficacy and safety of infliximab in the treatment of patients with ocular and neurological Behçet’s disease refractory to standard immunosuppressive drugs. Rheumatol Int 2011;31(1):33–7. 19859715 10.1007/s00296-009-1213-z

[25] Hamuryudan V Seyahi E Ugurlu S Melikoglu M Hatemi G Ozguler Y Pulmonary artery involvement in Behçet’s syndrome: Effects of anti-Tnf treatment. Semin Arthritis Rheum 2015;45(3):369–73. doi:10.1016/j.semarthrit.2015.06.008 26190564

[26] Emmi G Bettiol A Silvestri E Di Scala G Becatti M Fiorillo C Vascular Behçet’s syndrome: an update. Intern Emerg Med 2019;14(5):645–52. doi:10.1007/s11739-018-1991-y 30499073

[27] Arida A Fragiadaki K Giavri E Sfikakis P. Anti-TNF Agents for Behçet’s Disease: Analysis of Published Data on 369 Patients. Semin Arthritis Rheum 2011;41(1):61–70. doi:10.1016/j.semarthrit.2010.09.002 21168186

[28] Remicade (infliximab) FDA Approval History. Drugs.com. Accessed February 15, 2025. https://www.drugs.com/history/remicade.html

[29] Suhler EB Smith JR Wertheim MS Lauer AK Kurz DE Pickard TD A Prospective Trial of Infliximab Therapy for Refractory Uveitis: Preliminary Safety and Efficacy Outcomes. Arch Ophthalmol 2005;123(7):903–12. doi:10.1001/archopht.123.7.903 16009830

[30] Kabul EG Yenil S Ulutas F Calik BB Cobankara V. Investigation of Biopsychosocial Status, Fatigue, Sleep Quality, Alexithymia, Cognitive Functions, and Quality of Life in Behçet’s Disease. Mediterr J Rheumatol 2023;34(4):436. doi:10.31138/mjr.290823.ib 38282923 PMC10815526

[31] Hibi T Hirohata S Kikuchi H Tateishi U Sato N Ozaki K Infliximab therapy for intestinal, neurological, and vascular involvement in Behçet disease: Efficacy, safety, and pharmacokinetics in a multicenter, prospective, open-label, single-arm phase 3 study. Med U S 2016;95(24). doi:10.1097/MD.0000000000003863 PMC4998455 27310969

[32] Seyahi E. Behçet’s disease: How to diagnose and treat vascular involvement. Best Pract Res Clin Rheumatol 2016;30(2):279–295. doi:10.1016/j.berh.2016.08.002 27886800

[33] Kehribar D Ozgen M. Infliximab treatment in refractory vascular Behçet’s disease: A single-center experience. VASCULAR 2020;28(6):829–833. doi:10.1177/1708538120927701 32448079

[34] Aksoy A Yazici A Omma A Cefle A Onen F Tasdemir U Efficacy of TNFα inhibitors for refractory vascular Behçet’s disease: A multicenter observational study of 27 patients and a review of the literature. Int J Rheum Dis 2020;23(2):256–261. doi:10.1111/1756-185X.13778 31976619

[35] Hatemi G Tukek NB Esatoglu SN Ozguler Y Taflan SS Uygunoglu U Infliximab for vascular involvement in Behçet’s syndrome. Clin Immunol 2023;253. doi:10.1016/j.clim.2023.109682 37385325

[36] Liu Y Wu J Ao Y Li L Wang X Zhang M The efficacy and safety of monoclonal anti-TNF antibodies in the treatment of vascular Behçet’s syndrome: A systematic review and meta-analysis. Autoimmun Rev 2025;24(9):103862. doi:10.1016/j.autrev.2025.103862 40602700

